# Preoperative anxiety as predictor of perioperative clinical events following carotid surgery: a prospective observational study

**DOI:** 10.1186/s13741-021-00223-2

**Published:** 2021-12-08

**Authors:** Manuela Aspalter, Florian K. Enzmann, Thomas J. Hölzenbein, Wolfgang Hitzl, Florian Primavesi, Lucia Algayerova, Patrick Nierlich, Christoph Kartnig, Reinald Seitelberger, Klaus Linni

**Affiliations:** 1 Department of Cardiac, Vascular and Endovascular Surgery, Paracelsus Medical University, Müllner Hauptstraße 48, 5020 Salzburg, Austria; 2 Research Office, Biostatistics, Paracelsus Medical University, Strubergasse 21, 5020 Salzburg, Austria; 3 Department of Ophthalmology and Optometry, Paracelsus Medical University Salzburg, Müllner Hauptstr. 48, 5020 Salzburg, Austria; 4 Research Program Experimental Ophthalmology and Glaucoma Research, Paracelsus Medical University, Muellner Hauptstrasse 48, 5020 Salzburg, Austria; 5 Department of Psychiatry, Division of Psychosomatic Medicine, Paracelsus Medical University, Müllner Hauptstraße 48, 5020 Salzburg, Austria

**Keywords:** Asymptomatic carotid artery stenosis, Carotid endarterectomy, Hospital anxiety and depression scale, Spielberger state and trait anxiety inventory

## Abstract

**Background:**

Psychological factors like anxiety and depression are recognised to play a causal role in the development of cardiovascular disease and they may also influence outcome after vascular surgery procedures. The aim of this study was to investigate the association of anxiety and depression with postoperative outcome following elective carotid surgery.

**Methods:**

Single centre prospective observational study of patients treated for asymptomatic carotid artery stenosis at an academic vascular surgery centre. Preoperative anxiety and depression were evaluated using self-reporting questionnaires: Spielberger State-Trait Anxiety Inventory (STAI-S/-T) and Hospital Anxiety and Depression Scale (HADS-A/-D). Postoperative morbidity and mortality were assessed with the primary composite endpoint of stroke, myocardial infarction (MI) and death. Standard reporting guidelines for carotid disease were applied.

**Results:**

From June 2012 to November 2015, 393 carotid endarterectomies (CEA) were performed at our institution. Out of those, 98 asymptomatic patients were available for analysis (78% male; median age, 71.1 years). Median scores of self-reporting questionnaires did not differ from published data of the general population (STAI-T, trait component, median, 36; IQR, 31-42.75; STAI-S, state component, median, 38; IQR, 32-43; HADS-A median, 6; IQR, 3-8; HADS-D median, 4; IQR, 2-7). Cardiovascular risk factors were similar in anxious and non-anxious patients. The composite endpoint of stroke, MI and death occurred significantly more often in patients presenting with a preoperative HADS-A score higher than 6 (10.5%, 95% CI, 3-25; *p* =.020).

**Conclusions:**

The present study indicates that preoperative anxiety is associated with the occurrence of intra- and postoperative neurological events in patients undergoing CEA. Patients who had a preoperative HADS-A score of 6 or less had a very low probability of experiencing these complications.

## Background

Convincing evidence strengthens the hypothesis that psychosocial factors influence the development of somatic pathophysiological changes through several pathways. So far, identified pathways include dysregulation of the pituary-adrenal axis, sympathoadrenal hyperactivity, changes in the activity of the autonomic nervous system, alterations in platelet receptors and reactivity and modifications of the immune system. Recent studies indicate that mood disturbances such as anxiety and depression play a causal role in the development of cardiovascular disease (Bomhof-Roordink et al. 2015; Daskalopoulou et al. 2016; Kubzansky et al. 2006; Matthews et al. 1998; Narita et al. 2007; Nyrønning et al. 2019). Furthermore, these factors have been demonstrated to influence postoperative outcome following cardiac and general surgery (Blumenthal et al. 2003; Rosenbloom et al. 2009; Szekely et al. 2007; Tully et al. 2008; Villa et al. 2020).

Bearing the potential risk of stroke, carotid artery surgery is particularly stressful for patients. Outcome may therefore be influenced by psychological factors as well. Health-related quality of life has been assessed in patients undergoing surgical and endovascular procedures for atherosclerotic stenosis of the internal carotid artery (ICA) (Attigah et al. 2011; Cohen et al. 2011; Pearson et al. 2005; Stolker et al. 2010). However, the correlation of preoperative mood state and postoperative outcome has never been investigated. The aim of this study was to examine the influence of psychological factors, specifically anxiety and depression, on postoperative outcome after elective, surgical ICA revascularisation.

## Methods

### Study population

**Fig. 1:**
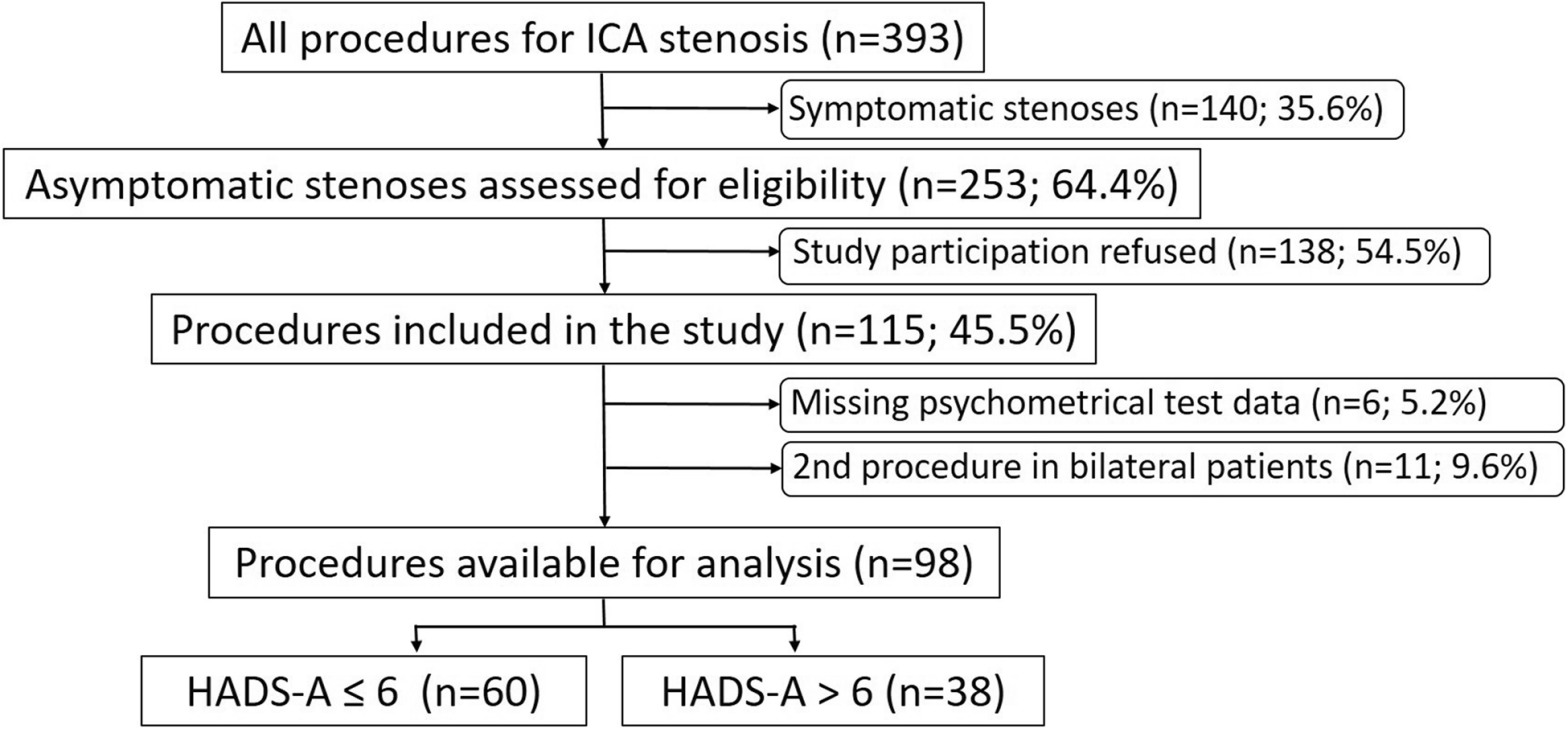
CONSORT diagram of patients undergoing carotid endarterectomy between June 2012 and November 2015. ICA, internal carotid artery

All consecutive patients undergoing revascularisation for high-grade ICA stenosis were prospectively evaluated and assessed for inclusion in this study (see Fig. 1).

High-grade stenosis was defined as lesions > 70% stenosis, calculated according to the NASCET guidelines (North American Symptomatic Carotid Endarterectomy Trial 1991). Patients were classified asymptomatic when no neurological symptoms (stroke, amaurosis fugax or transient ischemic attack) involving the ipsilateral carotid territory occurred within 180 days prior to the procedure (Naylor et al. 2018). All patients were pre- and postoperatively evaluated by an independent neurologist and ENT (ear nose throat) specialist, who were both blinded in terms of psychological test results, to identify any changes in neurological or laryngeal status.

Patients who underwent staged bilateral ICA revascularisation where included in the study. The first surgical intervention was considered and subjected to further analysis. Follow-up was recorded for both sides with special emphasis on the exact discrimination of the side of interest. Results of the contralateral side of bilateral patients were not subjected to analysis, since every patient could only be counted once.

The study was conducted in accordance with the principles of the Declaration of Helsinki and Good Clinical Practice guidelines. All participants provided written informed consent before inclusion in the study. The local ethics committee was informed but waved approval due to the descriptive design of the study.

All patients operated for recently symptomatic ICA stenosis, redo-procedures or previously diagnosed with a personality disorder were excluded from the study. Reporting guidelines for carotid disease were applied (Timaran et al. 2011).

### Depression and anxiety assessment

Psychometrical assessment was performed using the Spielberger State and Trait Anxiety questionnaire (STAI-S and STAI-T) (Spielberger et al. 1970) and the Hospital Anxiety and Depression Scale (HADS) (Zigmond et al. 1983). Both questionnaires are self-report measures, which were previously used with cardiovascular patients (Aleksic et al. 2006; Attigah et al. 2011; Szekely et al. 2007). Patients completed the questionnaires 24 h before and 24 h, 30 days and 1 year after the procedure. The STAI contains 20 statements relevant to generalised (trait component) and 20 statements relevant to current (state component) anxiety symptoms. Patients select the answers, which reflect their situation best (1-4 points). Therefore, possible scores range from 20 to 80 for each component. In the cardiac surgery literature, a cut-off value of 45 points was previously used (Szekely et al. 2007). The HADS score contains 14 statements relevant to generalised anxiety (HADS-A) and depression (HADS-D). Each of the subscales consists of 7 statements, which can be answered on a four-point Likert scale (0-3). Therefore, possible scores range from 0 to 21 for each subscale. For the HADS questionnaire, no generalizable cut-off values exist. As an orientation, scores from 0-7 in either subscale are rated normal, from 8-10 intermediate and 11 or higher indicate the possible presence of anxiety and depression as a mood disorder as suggested in the original publication (Zigmond et al. 1983).

### Surgical technique

Patients underwent ICA revascularisation using either eversion technique or conventional endarterectomy with patch plasty according to surgeons’ preference. Eversion endarterectomy under locoregional anaesthesia (LA), which consisted of both deep and superficial cervical plexus block, represents the standard procedure in our institution. Only in selected cases, conventional endarterectomy under LA or general anaesthesia (GA) and shunt placement was performed. Contraindications for LA included claustrophobia, severe hypacusis and severe language difficulties. The length of the whole surgical procedure and carotid cross clamping times were registered. Twenty-four hours postoperatively, all patients underwent close blood pressure and heart rate monitoring in an intermediate care unit.

### Clinical evaluation, follow-up surveillance

Morbidity and mortality were recorded. In order to account for all major adverse events, the composite of stroke, myocardial infarction (MI) and death (30-day mortality) was selected as primary endpoint. Secondary endpoints were major bleeding, surgical site infection, hoarseness, dysphagia and cranial nerve injuries.

On postoperative day one, a neurologist and ENT specialist examined all patients for early postoperative complications. Neurologic events were defined as any new neurological symptoms involving the ipsilateral carotid territory, with or without computed tomographic scan changes. With regard to MI, in case symptoms like chest pain occurred, electrocardiography was performed and cardiac enzymes were evaluated.

In addition to regular outpatient clinic visits with physical examination and colour-coded duplex ultrasound of the ICA 30 days, 6 month, 1 year after the procedure and yearly thereafter, the patients were asked to complete the self-reporting questionnaires 30 days and 1 year following the procedure. Patients who did not attend their regular outpatient clinic visits were contacted by telephone. In the rare circumstance, the patient could not be reached, his or her general practitioner (GP) was called for further information.

### Statistical analysis

Data were checked for consistency and normality. Fisher’s exact test or Pearson’s test were used to analyse cross tabulations and independent Student *t* tests were used to test means. Logistic regression models were used. For the combined endpoint stroke, MI or death with HADS-A as predictor, a logistic regression was applied and a cut-off for predicting patients free of the combined endpoint was selected. All reported tests were two-sided, and *p* values < .05 were considered as statistically significant. All statistical analyses in this report were performed by use of STATISTICA 13 (Hill, T. & Lewicki, P. Statistics: Methods and Applications. StatSoft, Tulsa, OK) and SPSS 24 (IBM SPSS Statistics for Windows, Version 21.0., Armonk, NY).

## Results

### Patients’ characteristics

From June 2012 to November 2015, a total of 393 carotid endarterectomies were performed at our institution. Of those, 98 patients were available for analysis (78% male; median age, 71.1 years; min, 46.3; max, 86 years) as depicted in Fig. 1. The logistic regression model suggested a HADS-A value ≤ 6 as cut-off for identifying patients free of the combined endpoint stroke, MI or death, and we therefore presented demographic data and clinical characteristics according to HADS-A values (Table 1). Cardiovascular risk factors were similar in patients in both groups.

**Table 1:** Demographic data and cardiovascular risk factors according to HADS-A values (*n* = 98)

	HADS-A ≤ 6 (%), *n* = 60 (61)	HADS-A > 6 (%), *n* = 38 (39)	*P* value
Male gender	49 (82)	27 (71)	.227^a^
Age, median (IQR)	72.2 (66.3-79.3)	69.9 (60.1-75.7)	.107^c^
Right side	31 (52)	14 (37)	.211^a^
BMI median (IQR)	26.7 (24.3-29.8)	26.8 (25.2-29.8)	.312^b^
< 25	19 (32)	9 (24)	.493^a^
25-30	32 (53)	20 (53)	1.0^a^
31-35	4 (7)	5 (13)	.303^a^
> 35	5 (8)	3 (8)	1.0^a^
Cardiovascular risk factors			
Smoking current	11 (19)	14 (38)	.056^a^
Smoking past	28 (48)	16 (43)	.677^a^
Hypertension	53 (88)	34 (89)	1.0^a^
Hyperlipidaemia	48 (80)	25 (66)	.154^a^
Diabetes	17 (28)	10 (26)	1.0^a^
Coronary artery disease	20 (33)	10 (26)	.507^a^
Peripheral artery disease	20 (33)	17 (45)	.289^a^
Decreased renal function	16 (27)	6 (16)	.320^a^
Family history	18 (31)	10 (27)	.819^a^

^a^Fisher exact

^b^Pearson’s chi-squared test, two-tailed

^c^Independent *t* test, *IQR* interquartile range

Living status, education, antidepressant medication and psychometrical scores are summarised in Table 2. A total of fifteen patients (15%) were on current antidepressant medication, with a trend towards more antidepressant use in anxious patients (HADS-A > 6, group 2), yet, not statistically significant (*p* = .068). More details on indication for surgery, ASA-classification, and procedural data are given in Table 3.

**Table 2:** Living status, education, antidepressant medication and psychometrical scores according to HADS-A values (*n* = 98)

	HADS-A ≤ 6 (%), *n* = 60 (61)	HADS-A > 6 (%), *n* = 38 (39)	*P* value
Living with another	47 (78)	27 (73)	0.626^a^
Basic education (primary school)	19 (32)	12 (33)	1.0^a^
Vocational training	31 (53)	19 (53)	1.0^a^
High school completed	4 (7)	3 (8)	1.0^a^
University completed	3 (5)	1 (3)	1.0^a^
Antidepressant medication	6 (10)	9 (24)	.068^a^
STAI-T median (IQR)	33 (29-38)	45 (38-48)	**< .001**^b^
STAI-T ≥ 45	0	17 (45)	
STAI-S median (IQR)	36 (30-39)	41 (38-49.5)	**< .001**^b^
STAI-S ≥ 45	7 (12)	15 (39)	
HADS-D median (IQR)	3 (1-5)	6.5 (3-9)	**< .001**^b^
HADS-D ≥ 9	2 (3)	12 (32)	

No data on living status in 3 patients, no data on education in 6 patients; *IQR* interquartile range, *STAI-T* Spielberger state and trait anxiety inventory-trait component, *STAI-S* Spielberger state and trait anxiety inventory-state component, *IQR* interquartile range, *HADS-D* hospital anxiety and depression scale-depression component

^a^Fisher exact

^b^Independent *t* test, two-tailed

**Table 3:** Indication for surgery, ASA-classification and procedural data according to HADS-A values (*n* = 98)

	HADS-A ≤ 6 (%), *n* = 60 (61)	HADS-A > 6 (%), *n* = 38 (39)	*P* value
Stenosis ipsilateral mean (IQR)	85.8 (80-90)	86.5 (80-90)	.551^a^
Stenosis contralateral mean (IQR)	45.3 (30-68.8)	41.4 (25-65)	.508^a^
ASA classification grade			
2	8 (17)	2 (7)	.301^b^
3	39 (81)	28 (93)	.188^b^
4	1 (2)	0	1.0^b^
Missing	12 (20)	8 (21)	.901^b^
Eversion endarterectomy under LA	55 (92)	34 (89)	.718^b^
Secondary general anaesthesia	0	2 (5)	.074^b^
Eversion endarterectomy under GA	3 (6)	1 (3)	0.568^b^
Conventional endarterectomy in LA	2 (3)	3 (9)	.322^b^
Secondary general anaesthesia	0	1 (3)	.211^b^
Shunt placement	2 (3)	3 (8)	.373^b^
Mean overall procedure time in minutes (IQR)	72 (57.8-82.3)	71 (55.8-83.8)	.897^a^
Mean ICA clamping time in minutes (IQR)	18 (11.8-22.0)	19 (11.5-25.5)	.507^a^

*ASA* American Association of Anaesthesiologist, *LA* locoregional anaesthesia, *GA* general anaesthesia, *ICA* internal carotid artery, *IQR* interquartile range

^a^Independent *t* test

^b^Fisher exact, two-tailed

### Psychometrical assessment

Median scores of self-reported questionnaires were in the normal range compared to published data on the general population. In brief, STAI-trait median, 36; IQR, 31-42.75; STAI-state median, 38; IQR, 32-43; HADS-A median, 6; IQR, 3-8; HADS-D median, 4; IQR, 2-7 (Table 2).

### Postoperative outcome

No perioperative MI, procedure related death or 30-day mortality was registered. Therefore, the primary endpoint (combined endpoint of stroke, MI and 30-day mortality) consisted of strokes, only.

**Fig. 2:**
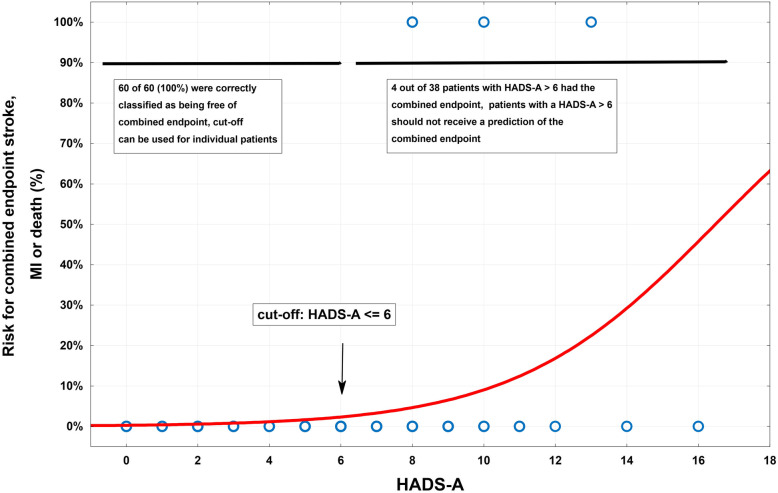
Risk for the combined endpoint of post-operative stroke, MI and death according to pre-operative HADS-A values. The cut-off ≤ 6 was selected for identifying patients free of the combined endpoint. Sixty patients fulfilled a HADS-A ≤ 6 and all 60 patients (100%) were correctly predicted of being free of the combined endpoint. Thirty-eight patients did not fulfil the cut-off and 4 out of 38 (10.5%) fulfilled the endpoint. If the cut-off is fulfilled, we suggest an individual prediction; however, if the cut-off is not fulfilled, no individual prediction should be made

The primary endpoint was seen significantly more often in patients who presented with a preoperative HADS-A score over 6 (group 2, 10.5%, 95% CI 3-25; *p* = .020). After applying the HADS-A cut-off ≤ 6, 60 (group 1, 61%) patients were identified being free of stroke, MI or death as illustrated in Fig. 2. All 60 patients (100%) were correctly predicted of being free of the combined endpoint. Four out of 38 patients (10.5%) with a HADS-A > 6 (all group 2) suffered from intraoperative ischemic neurological deficits (Table 4) and therefore fulfilled the combined endpoint. In 3 of these patients, the neurological symptoms resolved completely within 30 days of treatment, resulting in a modified Rankin score of 0 (Rankin et al. 1957). In the remaining patient, a minor contralateral weakness persisted, which did not affect his daily living as represented by a modified Rankin score of 1.

**Table 4:** Follow-up, minor and major complications according to HADS-A values (*n* = 98)

	HADS-A ≤ 6 (%), *n* = 60 (61)	HADS-A > 6 (%), *n* = 38 (39)	*P* value
Follow-up median in months (IQR)	55 (33-69)	59 (51-69)	.221^a^
Bleeding	1 (2)	1 (3)	1.0^b^
Infection	1 (2)	1 (3)	1.0^b^
Hoarseness	13 (22)	13 (34)	.240^b^
Dysphagia	1 (2)	1 (3)	1.0^b^
Cranial nerve injuries	5 (8)	5 (13)	.504^b^
Recurrent laryngeal nerve palsy, transient	2 (3), 2 (100)	5 (13), 4 (80)	.105^b^
Hypoglossal nerve palsy, transient	3 (5), 2 (67)	0	.280^b^
Stroke	0	4 (11)	**.020**^b^
Minor stroke (modified Rankin score 0-1)		4 (100)	
Myocardial infarction	0	0	1.0^b^
30-day mortality	0	0	1.0^b^
Overall mortality	9 (15)	3 (8)	.27^b^
Lost to follow-up	4 (7)	1 (3)	.381^b^

^a^Fisher exact

^b^Independent *t* test, two-tailed, *IQR* interquartile range

With regard to other peri- and postoperative complications, no association with the investigated self-reported scores was observed; for more details, see Table 4.

Five patients were lost to follow-up as indicated in Table 4, including an 81 year-old man 1.5 months after surgery, a 68-year-old man 4 months following surgery, a 76-year-old man 6 months following surgery, a 57-year-old man 7 months after surgery and a 59-year-old woman who was last seen 10 months following her first surgery. In terms of preoperative HADS-A values, 4 of these patients had a value of 6 or less (group 1) and one patient a value of more than 6 (group 2).

**Fig. 3:**
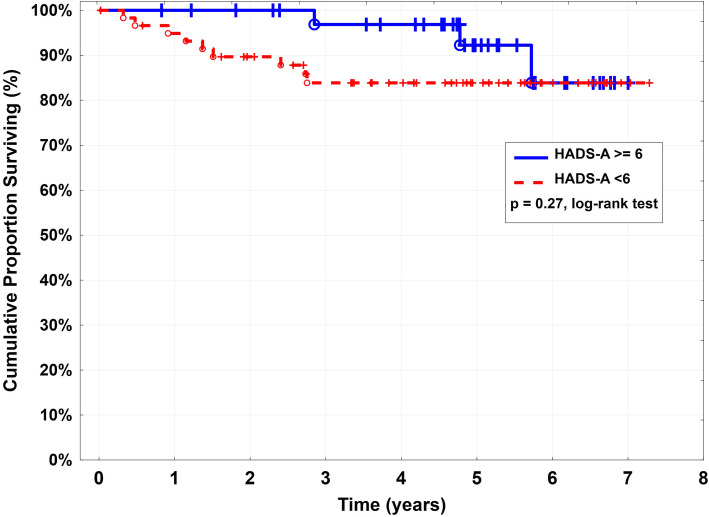
Kaplan-Meier estimations for survival according to preoperative HADS-A values

During follow-up, 12 patients died (median age 74.5 years; IQR, 67.8-83.8), median time from surgery 2 years (IQR, 1.09-2.78) (Fig. 3). The cause of death was unknown in four patients, three died of cancer (pulmonary cancer in 2, genitourinary cancer in 1) and four due to cardiogenic shock. One woman died of fatal contralateral stroke 5.7 years after carotid surgery.

## Discussion

This study indicates, for the first time, that preoperative anxiety increases the risk to develop perioperative neurological events in carotid endarterectomy (CEA) patients.

Self-rating questionnaires were infrequently used in carotid surgery patients. To the best of our knowledge, correlations of preoperative anxiety and postoperative outcome have never been performed. Pearson and colleagues found a correlation of higher pre-operative state-anxiety and poorer mental functioning using the MOS 36-item Short-Form Health Survey in 39 patients (Pearson et al. 2005) This correlation, however, was unrelated to complications or physical functioning; therefore, reporting bias in anxious people was suspected. In their prospective study investigating postoperative cognitive function after CEA, Aleksic and colleagues used the HADS questionnaire to quantify anxiety and depression in their study population (Aleksic et al. 2006). They compared HADS values from CEA patients (*n* = 33) and patients undergoing surgery for arterial occlusive disease (*n* = 25). In CEA, patient’s depressive symptoms (HADS-D) diminished postoperatively and then increased again at follow-up. Anxiety scores (HADS-A), on the other hand, declined over time. These trends, however, were not statistically significant. Interestingly, HADS-A values of patients undergoing surgery for arterial occlusive disease increased significantly at follow-up compared to baseline evaluations. In their comparably small study population, no correlation with post-operative complications was performed. Investigating patients’ satisfaction in CEA patients under local anaesthesia (*n* = 102), Attigah et al. found reduced satisfaction in patients with increased anxiety and depression, demonstrated by a HADS-A score of ≥ 11 and an HADS-D score of ≥ 9 (Attigah et al. 2011). Minor complications with plexus anaesthesia, however, reduced patients’ satisfaction as well. In a sub-analysis of the SAPPHIRE-Trial, Stolker and colleagues found that patients reported significantly less neck pain, less difficulty swallowing, less difficulty driving and higher scores at the SF-36 questionnaire after carotid artery stenting (CAS) compared to CEA at 2 weeks (Stolker et al. 2010). These findings favouring CAS, however, resolved at 1 month postoperatively. Cohen and colleagues, who performed a sub analysis of the CREST trial, found similar results (Cohen et al. 2011).

In our study, anxiety was assessed using two different self-report measures: the HADS questionnaire and the STAI. The HADS questionnaire has been used in CEA patients before, even though, no validation in this specific patient population has been performed. In the most recent comprehensive review on the clinical performance of the HADS instrument, Bjielland and colleagues found a large variety in optimal cut-off values dependent on populations screened (Bjelland et al. 2002). As an orientation, Johnson and colleagues (Johnson et al. 1995) studied post stroke patients (*n* = 93) and their optimal cut-off scores were estimated to be 5+ for HADS-A and 4+ for HADS-D with significantly lower specificity for both anxiety and depression (.46 and .44, respectively) (Bjelland et al. 2002).

The choice of the cut-off of the HADS-A instrument was based on empirical observations as well as on the results of the logistic regression model. The main objective of this cut-off was to identify patients free of the combined endpoint of stroke, MI and death. Two patients with a preoperative HADS-A value of 8 fulfilled this endpoint; hence, a cut-off of 8 or more was excluded. There was no patient experiencing the combined endpoint with a HADS-A score of 7. Nevertheless, this cut-off was not selected, because the selection of such a cut-off underlies various random effects, e.g. sample bias. In order to improve the prediction power of this endpoint, the cut-off of 6 was finally selected. Sixty fulfilled a HADS-A ≤ 6 and all 60 patients were correctly predicted of being free of the combined endpoint. We strongly suggest that the proposed cut-off of HADS-A values of more than 6 for CEA patients should be examined in other studies to better judge the prediction power for identifying patients free of the combined endpoint. Please note that if a patient presents with a preoperative HADS-A score of more than 6, this does, of course, not necessarily imply that the endpoint is going to be fulfilled. Only the risk is increased as illustrated in Fig. 2 and further individual decisions can be made.

In the present study, only asymptomatic patients with high-grade atherosclerotic carotid artery stenosis (> 70% according to NASCET criteria) were included to reduce reporting bias and ensure homogeneity of the study population (North American Symptomatic Carotid Endarterectomy Trial. 1991). Symptomatic patients, who by definition experienced neurological symptoms previously, may be particularly anxious and therefore, preoperative self-reporting scores may overestimate their anxious state. To date, no validation of these psychometric tests in atherosclerotic carotid artery stenosis patients regardless of their neurological state (asymptomatic/symptomatic) exists. Investigating symptomatic patients in the setting of a prospective study would definitely broaden our knowledge in this field. Therefore, further research is required.

With regard to postoperative complications, the crude numbers in this study are high compared to published data on asymptomatic CEA procedures (Cui et al. 2018) and when symptomatic and asymptomatic patients were combined (Chou et al. 2016). A neurologist examined all patients before and 1 day after the procedure. As a consequence, even minor postoperative changes were identified. These circumstances may be the reason, why we detected so many neurological events. Thorough postoperative assessment was completed by an evaluation conducted by an ENT specialist with special emphasis on laryngeal nerve function (fiberoptic laryngoscopy).

The most substantial limitation of the present study is its small sample size. It may have been underpowered to detect more severe differences in postoperative complications to draw strong and broadly applicable conclusions.

Further psychiatric assessment of the patients to better characterise their anxious state would have increased our knowledge in the setting of this prospective study. On the contrary, additional psychiatric evaluation may influence patients’ preoperative mood and therefore we decided to only use the self-rating questionnaires as performed by others (Szekely et al. 2007; Tully et al. 2008). No in-depth assessment of further intraoperative use of anxiolytic medication was performed. However, all procedures were performed in accordance with surgical and anaesthesiological standard techniques in all patients. The preoperative HADS-A values were neither known by the operating surgeon nor by the anaesthesiologist, the neurologist or ENT specialist; therefore, detection bias is highly unlikely.

The present study suggests that future patients be assessed for anxiety to estimate their individual risk for neurological events during carotid surgery. Additionally, interventions aimed at reducing anxiety in highly anxious patients should be subjected to further research to elucidate their potential to further reduce stroke risk in this particular patient cohort (Vasdekis et al. 2015; Villa et al. 2020). Conceivably, anxious patients may benefit from relaxation techniques such as relaxation breathing, muscle progressive relaxation, guided imagery, music therapy, or hypnosis as suggested before (Nilsson et al. 2009; Vasdekis et al. 2015; Villa et al. 2020). Furthermore, administration of anxiolytic medication prior to surgery or change of anaesthetical regimen to general anaesthesia may be beneficial. This aspect, however, was not investigated in the present study and should be subjected to further research.

## Conclusions

Despite its limitations, this study adds further knowledge regarding the contribution of psychosocial factors on postoperative morbidity after carotid artery surgery. Moderate preoperative anxiety was independently associated with the occurrence of perioperative neurological events. Further prospective studies with larger patient cohorts are warranted to increase our understanding of the underlying pathophysiological mechanisms.

## Data Availability

The datasets used and/or analysed during the current study are available from the corresponding author on reasonable request.

## Abbreviations

ASA: American Association of Anaesthesiologist
CAS: Carotid artery stenting
CEA: Carotid endarterectomy
CNI: Cranial nerve injuries
GA: General anaesthesia
HADS-A: Hospital anxiety and depression scale-anxiety component
HADS-D: Hospital anxiety and depression scale-depression component
ICA: Internal carotid artery
IQR: Interquartile range
LA: Locoregional anaesthesia
MI: Myocardial infarction
STAI-S: Spielberger state and trait anxiety inventory-state component
STAI-T: Spielberger state and trait anxiety inventory-trait component
